# Temporal evolution of laboratory characteristics in patients critically ill with COVID‑19 admitted to the intensive care unit (Review)

**DOI:** 10.3892/mi.2023.112

**Published:** 2023-09-14

**Authors:** Stelios Kokkoris, Angeliki Kanavou, Panagiotis Kremmydas, Dimitrios Katsaros, Stavros Karageorgiou, Aikaterini Gkoufa, Vasiliki Epameinondas Georgakopoulou, Demetrios A. Spandidos, Charalampos Giannopoulos, Marina Kardamitsi, Christina Routsi

**Affiliations:** 1 First Department of Intensive Care, Evangelismos Hospital, Medical School, National and Kapodistrian University of Athens, 10676 Athens, Greece; 2 Department of Infectious Diseases, Laiko General Hospital, Medical School, National and Kapodistrian University of Athens, 11527 Athens, Greece; 3 Laboratory of Clinical Virology, School of Medicine, University of Crete, 71003 Heraklion, Greece

**Keywords:** coronavirus, COVID-19, intensive care unit, laboratory values, clinical chemistry, trajectory, evolution, temporal changes, mortality

## Abstract

In the context of coronavirus disease 2019 (COVID-19), laboratory medicine has played a crucial role in both diagnosis and severity assessment. Although the importance of baseline laboratory findings has been extensively reported, data regarding their evolution over the clinical course are limited. The aim of the present narrative review was to provide the dynamic changes of the routine laboratory variables reported in patients with severe COVID-19 over the course of their critical illness. A search was made of the literature for articles providing data on the time-course of routine laboratory tests in patients with severe COVID-19 during their stay in the intensive care unit (ICU). White blood cell, neutrophil and lymphocyte counts, neutrophil to lymphocyte ratio, platelet counts, as well as D-dimer, fibrinogen, C-reactive protein, lactate dehydrogenase and serum albumin levels were selected as disease characteristics and routine laboratory parameters. A total of 25 research articles reporting dynamic trends in the aforementioned laboratory parameters over the clinical course of severe COVID-19 were identified. During the follow-up period provided by each study, the majority of the laboratory values remained persistently abnormal in both survivors and non-survivors. Furthermore, in the majority of studies, the temporal trends of laboratory values distinctly differentiated patients between survivors and non-survivors. In conclusion, there are distinct temporal trends in selected routine laboratory parameters between survivors and non-survivors with severe COVID-19 admitted to the ICU, indicating their importance in the prognosis of clinical outcome.

## 1. Introduction

The coronavirus disease 2019 (COVID-19) pandemic, caused by severe acute respiratory syndrome coronavirus-2 (SARS-CoV-2), rapidly spread throughout China and became a worldwide public health crisis (1). Initially considered a respiratory disease, capable of inducing acute hypoxemic respiratory failure, COVID-19 is often complicated by multi-organ dysfunction syndrome, affecting almost all organs in its most severe forms (2,3).

Laboratory medicine, apart from its crucial contribution to the etiological diagnosis of SARS-CoV-2 infection, using reverse transcription-polymerase chain reaction, also played a critical role in assessing the severity of the disease. Certain abnormalities in laboratory parameters at the onset of the disease were identified early during the pandemic, including blood count distribution, coagulation profiles, and markers of systemic inflammation and tissue damage. The critical role of laboratory medicine has been demonstrated in publications, including research articles and literature reviews (4-9), presenting the most characteristic laboratory abnormalities observed in patients with severe COVID-19, particularly those admitted to the intensive care unit (ICU). However, the majority of these studies focused on the initial values of the laboratory variables. As a result, the available information on the trajectory of the laboratory values during the stay in the ICU and its association with outcomes is limited. The present narrative review aimed to provide evidence from the current literature regarding the temporal evolution of the most characteristic hematological and biochemical parameters, which are routinely tested in patients admitted to the ICU due to COVID-19, over the course of the disease.

## 2. Data collection methods

In order to identify studies including the analyses of the temporal evolution of laboratory test parameters, which are routinely measured in patients admitted to the ICU due to COVID-19, a search was performed of the PubMed and Google Scholar databases using the keywords ‘COVID-19’, ‘SARS-CoV-2’, ‘critical care’, ‘ICU’, ‘mechanical ventilation’, ‘coronavirus disease 2019’, ‘evolution’, ‘trajectory’, ‘hematological’, ‘biochemical’, ‘mortality’, for articles in the English language published between January, 2020 and September, 2022. The reference lists of the selected articles were also searched, as well as the citation articles for relevant publications. The following data were extracted: The first author, country, year of publication, sample size, and the hematological and biochemical parameters in the included articles. White blood cell (WBC), neutrophil and lymphocyte counts, neutrophil to lymphocyte ratio (NLR), platelet counts, as well as the levels of D-dimer, fibrinogen, C-reactive protein (CRP), lactate dehydrogenase (LDH) and serum albumin were included as selected variables of interest. The selection of the abovementioned laboratory variables was based on their characterization as unique laboratory characteristics of COVID-19, as well as on their routine measurement for monitoring purposes during the stay in the ICU.

## 3. Results of the literature search

Following a comprehensive search and review, 25 research articles, reporting the dynamic trends of the marked laboratory tests of severe COVID-19 over the disease progress were identified, as well as a review article including earlier studies from China, describing the temporal changes of hematological findings in a general population with COVID-19, not exclusively in patients in the ICU (10). The main results of the present narrative review are summarized in Table I. Detailed information on the evolution of the main laboratory abnormalities over the clinical course in the ICU, as well as their impact on clinical outcomes is presented in detail below.

### Hematological parameters. WBC and neutrophil counts

An elevated WBC count may be the result of various inflammatory responses, including both bacterial and viral infections (11). In the context of COVID-19, although the association between a high WBC count upon admission and the increased severity and mortality has been widely reported (12-14), only a limited number of articles provide information on the progression of WBC count during the stay in the ICU (15-22).

Even in early reports, the WBC count was shown to remain at high levels, peaking on day 10 of illness (15) or on day 7 following admission (16), while decreasing at the time of discharge only in less severe cases (17). In an early cohort study from Wuhan, China (18), the WBC count was shown to be significantly higher in non-survivors compared to survivors for 19 days following disease onset. On the other hand, in a study including 548 patients with COVID-19 from a national cohort in China, an increased neutrophil count exhibited an upward trend in non-survivors, whereas the neutrophil count was stable or exhibited a downward trend in survivors (19). In a large European-based cohort providing the almost real-time assessment of 639 patients critically ill due to COVID-19, the WBC count increased over time, peaking between days 2 and 3 following admission to the ICU (20). More specifically, in non-survivors, the WBC count was persistently higher during the first 7 days of ICU stay (20). Furthermore, in the multicenter study by Zanella *et al* (21), including 1,260 patients critically ill with COVID-19, the WBC count upon admission to the ICU was higher in non-survivors, whereas the neutrophil count was not. However, the trend in the WBC count during the stay in the ICU did not differ significantly between the survivors and non-survivors (21). By contrast, in a study from New York, USA identifying clinical markers that demonstrated a temporal progression associated with mortality, the WBC count was found to gradually increase prior to mortality (22).

*Lymphocyte count.* Absolute lymphopenia has been recognized as a common feature, particularly in patients with severe COVID-19, associated with an increased risk of developing adverse outcomes (5,21). Dynamic changes in the lymphocyte count over time have been reported in a limited number of studies, as indicated below.

In the large multicenter study by Xie *et al* (23), persistent lymphopenia was observed in both survivors and non-survivors during the first 14 days in the ICU, whereas in another study (24), time-dependent analysis revealed that the lymphocyte counts differed significantly between survivors and non-survivors, although no significant difference was observed over time. Persistent lymphopenia was also reported by Zhou *et al* (25) during the first 3 weeks following the onset of critical illness, and this was more profound in non-survivors. By the day of discharge, lymphocytes had returned to normal levels only in the less severe cases. Additional data have also revealed marked lymphopenia over time, which is more severe in non-survivors (17,18,26,27), suggesting that this variable may be used as a laboratory marker to distinguish patients with COVID-19 who are at a high risk of mortality at any time point during their clinical course. In another study by Chen *et al* (19), when addressing the dynamic changes in different biomarkers, the survivors were found to exhibit an increasing trend for lymphocytes (1.2-fold in midterm, and 1.4-fold at the end of hospitalization); in non-survivors, the lymphocytes remained at low levels without a noteworthy increase (19). Finally, in the large study by Zanella *et al* (21), daily values of lymphocytes were associated with survival; however, no significant difference was found in the progression of the lymphocyte count as a prognostic marker for outcomes.

*NLR.* The NLR, calculated by dividing the absolute neutrophil count by the absolute lymphocyte count, is considered to be linked to innate immunity, and serves as a useful biomarker for the evaluation of systemic inflammatory responses in patients with sepsis (28). An increased baseline NLR has been well-described in severe COVID-19 (18,29-33) and has also been found to be associated with poor outcomes (34,35). The evolution of NLR over the clinical course of severe COVID-19 has been analyzed in the studies discussed below.

In an early retrospective study from China (36), an increasing trend in the NLR in non-survivors, significantly differed compared with that in survivors. In addition, the peak value during hospitalization was higher in non-survivors, indicating a role of the NLR time course in the risk of mortality. Similarly, Zanella *et al* (21) reported an increased NLR in non-survivors at the time of admission to the ICU, as well as during the stay in the ICU. Both daily values and the slope of the ratio over time were associated with survival. In accordance, in the study by Wendel-Garcia *et al* (20), the NLR was persistently increased and was significantly higher in non-survivors in the ICU compared to survivors during the first 7 days in the ICU. A persistently high NLR was also observed in a small study, including 24 patients with COVID-19 in the ICU, peaking on day 10 of illness (15).

By using the maximum value of NLR during the first 3 days after being diagnosed with severe COVID-19, Ma *et al* (37) reported the ability of this biomarker to discriminate patients with moderate-severe acute respiratory distress syndrome eligible for veno-venous extracorporeal membrane oxygenation. Finally, in the study by Chen *et al* (19), NLR along with leukocytes, and other laboratory variables were maintained at significantly lower levels or exhibited a slight downward trend in survivors. By contrast, the NLR exhibited an upward trend or maintained higher levels in non-survivors.

*Platelet count.* Platelets are key regulators of thrombosis, inflammation and immunity and as such, they contribute to the pathophysiology of COVID-19 and the development of COVID-19-associated complications (38,39). Moreover, an abnormal platelet count, particularly thrombocytopenia, is relatively common in patients in the ICU (40). Temporal changes in the platelet count over the course of severe COVID-19 have been reported in a limited number of studies.

A previous study demonstrated that the trajectory of the platelet count during 28 days of hospitalization for patients with critical illness due to COVID-19 was persistently lower in critically ill patients and in non-survivors (39). Consistently, other studies have demonstrated that the platelet count is significantly decreased in non-survivors and the temporal changes in the levels of this marker differ markedly between survivors and non-survivors throughout the clinical course (26,41).

In a study on the coagulation profiles of critically ill patients due to COVID-19 stratified by a sequential organ failure assessment (SOFA) score >10 or ≤10(42), the platelet count increased from baseline to day 14 in both groups; a lower count was observed over time in patients with a SOFA score >10 compared to those with a score ≤10(42). The platelet count was increased in all patients, with ICU survivors presenting consistently higher counts during the first 7 days in the large cohort study by Wendel-Garcia *et al* (20). In another study, the median values of platelets were maintained within the normal range in survivors, significantly higher than those in non-survivors among 195 critically ill patients with COVID-19 over the course of days 1 to 28(25). However, the platelet count was not independently associated with mortality in the multivariable analysis. Similarly, in another study (18) the survivors exhibited a significantly higher level of platelets upon admission and an increasing trend during hospitalization, whereas non-survivors had a lower level of platelets upon admission, which further decreased afterwards.

*D-dimer levels.* D-dimer, a fibrin degradation product, represents a non-specific acute phase reactant, the levels of which may be elevated in acute inflammatory illnesses, as well as in venous thromboembolism. In the setting of COVID-19, elevated values of D-dimer are common, particularly in severe cases. However, the clinical relevance of elevated D-dimer levels is multifaceted. Increased D-dimer values may reflect disease activity and cannot be solely attributed to venous thromboembolic complications (43). Various studies have confirmed elevated baseline D-dimer levels as a predictor for both mortality and complications. However, the dynamic trend of D-dimer levels in patients with COVID-19 has been analyzed in a limited number of studies, as described below.

In a previous study, the D-dimer level, although not different upon admission, exhibited an increase over time in non-survivors in an early study from China (18), as well as in another study from Italy (44). Similarly, in another study significant dynamic changes in D-dimer levels over the first 14 days in the ICU were observed in non-survivors (23). In the large study by Wendel-Garcia *et al* (20), the D-dimer levels remained elevated throughout the first 7 days in the ICU in patients with unfavorable outcomes. In another small study (45) evaluating the coagulation function in 40 patients with COVID-19, the D-dimer values on days 5 and 10 were persistently high, although lower than those on the day of admission. Dynamic changes in the D-dimer levels in 577 patients admitted to the ICU due to COVID-19 were also observed in another study; these levels were higher and a higher increase rate was observed in non-survivors compared with survivors, indicating the utility of dynamic changes of D-dimer levels (46). In addition, in a study aiming to predict the development of venous thromboembolism in patients critically ill with COVID-19, the median D-dimer levels significantly increased on days 4 and 8 post-ICU admission in the patients who developed thromboembolism compared to those who did not (47).

A previous study demonstrated that D-dimer levels, measured every other day after admission, were persistently higher in mechanically ventilated patients with COVID-19 compared to those under non-invasive ventilation (16). Finally, another study also demonstrated the dynamic profile of coagulation parameters, tracked from days 1 to 14 after admission at 3-day intervals, in 183 patients with COVID-19, and revealed significantly higher D-dimer levels in non-survivors compared to survivors upon admission, as well as in late hospitalization (48). However, in another study (17), although higher D-dimer values upon ICU admission were observed in non-survivors compared to survivors, the change from admission to the 7th day did not differ between them. In accordance, in the large study by Zanella *et al* (21) evaluating the daily values and trends over time of relevant laboratory parameters in 1,260 patients in the ICU with COVID-19, the D-dimer values upon admission to the ICU were higher in non-survivors than in survivors; these values then decreased in both groups, but were not associated with survival in joint modeling. Similarly, elsewhere, although it was the single largest identifier of COVID-19 status, the D-dimer trajectory was not associated with outcomes (49).

*Fibrinogen.* Fibrinogen is a key coagulation factor in the common pathway of the coagulation cascade. It is an acute phase protein with a high molecular weight and is a known key regulator of inflammation (50). In the context of COVID-19, the plasma levels of fibrinogen are frequently increased and are associated with excessive inflammation, disease severity and ICU admission. To date, the temporal trends of fibrinogen levels over the course of the disease have only rarely been described in patients with COVID-19 in the ICU.

In the early study by Corrêa *et al* (42), reporting laboratory tests at baseline and on days 1, 3, 7 and 14 in patients stratified by a SOFA score >10 or ≤10, the highest fibrinogen values were observed on day 1 in the group with a SOFA score ≤10 and on day 3 in the group with a SOFA >10. On day 14, a more pronounced decrease in plasma fibrinogen levels was observed in patients with a SOFA score ≤10(42). In another study, the fibrinogen levels, although persistently high, did not differ between survivors and non-survivors both upon admission and on day 7(17). Consistently, individual trajectories for fibrinogen values did not exhibit any association with mortality in a study evaluating coagulation and endothelial function biomarkers in 14 patients with COVID-19(49). Finally, it was also previously demonstrated that the fibrinogen plasma levels were persistently high over a follow-up period of 20 days after ICU admission in patients with COVID-19 either with or without the diagnosis of venous thromboembolism (47).

### Biochemical parameters. LDH

LDH is a cytoplasmic enzyme which is crucial for cellular energy production. LDH plasma concentrations increase upon cell injury as a result of inflammation or hypoxia. In the context of COVID-19, elevated levels of LDH are considered to reflect lung and more widespread tissue damage. High levels of LDH upon admission have been used as a discriminatory laboratory marker for severity (5). The time course of LDH levels has been reported in a limited number of studies, as described below.

In an early report from Japan (15), LDH levels were elevated in the initial phase of illness and subsequently decreased in patients admitted to the ICU due to COVID-19. In the study by Chen *et al* (22), assessing the time course of various variables as a function of days to outcome, LDH levels exhibited a sharp change on the day of or a day prior to death, relatively to the values of survivors at the same time points. Persistently high LDH levels or increasing trends in LDH in non-survivors were reported in the large study by Wendel-Garcia *et al* (20), as well as in the study by Ouyang *et al* (26). Similarly, in another study by Xie *et al* (23), the LDH levels significantly decreased in survivors, but remained higher in non-survivors. Notably, in that study, the LDH levels, over time, exhibited significant differences between survivors and non-survivors (23). In addition, elsewhere, the time-dependent analysis of LDH levels reveled significant differences between survivors and non-survivors, as well as across time (24).

*CRP.* CRP, an acute phase serum protein, serves as a marker of the degree of inflammation. CRP has been identified as a key biomarker whose levels increase significantly in patients with severe COVID-19 and determines the progression of the disease (51-53). In a limited number of studies, the trend of CRP during the disease course has been reported, as described below.

In a small study from the first COVID-19 pandemic wave, CRP levels were elevated in the initial phase of illness and peaked on day 10 of illness (15). Consistently, longitudinal changes in CRP levels from the onset of symptoms exhibited similar trends in cases of either ICU admission or mortality (54).

In a European cohort of patients critically ill with COVID-19(20), CRP levels peaked on day 3 of stay in the ICU. In addition, ICU non-survivors were characterized by a significant increase in CRP levels, whereas the initial CRP levels did not differ between survivors and non-survivors. Similarly, in a multicenter study from North Italy (21) CRP levels at ICU admission were equally elevated in survivors and non-survivors, although continuously decreased over time in survivors. Τhe trends in CRP levels over the entire ICU stay were predictive of mortality (21). In another study, CRP levels decreased from admission to the 7th day of hospitalization in survivors, whereas an increase was observed in non-survivors (20). Moreover, in another study, the time dependent analysis of CRP levels did not reveal significant differences between survivors and non-survivors, although significant differences were observed over time (55); however, in another previous study, CRP values in non-survivors gradually increased with differences early or prior to mortality, relative to those of survivors (22). Finally, in the multicenter study by Wendel-Garcia *et al* (56), which provided the dynamics of the disease characteristics of patients critically ill with COVID-19 over the course of the pandemic, CRP levels during the first 5 days of ICU stay progressively displayed a more prominent decreasing trend over the duration of the pandemic. As regards the differences between survivors and non-survivors over time, CRP dynamics over the first days after ICU admission exhibited a more pronounced decline in non-survivors after the first pandemic wave, as compared to survivors (56).

It should be noted that dexamethasone and tocilizumab, both recommended for the treatment of severe COVID-19 since August, 2020(57) and January, 2021(58), respectively, are known to suppress the pro-inflammatory response, including CRP levels. Therefore, the more pronounced decrease in CRP levels during the stay in the ICU may reflect the systematic initiation of corticosteroids and/or tocilizumab. Zacharias *et al* (59) recently evaluated the effects of dexamethasone and tocilizumab on the trajectory of CRP levels among patients with critically ill COVID-19. Sequential CRP data were available in 174 patients receiving dexamethasone and in 40 patients receiving tocilizumab. Among the patients who received dexamethasone, CRP levels were significantly higher among the non-survivors. A significant reduction in CRP levels was observed in the first 3 days of treatment among the survivors and non-survivors, whereas over the subsequent week, the CRP levels increased among non-survivors, but not in survivors (59).

*Serum albumin.* Hypoalbuminemia upon hospital admission, possibly in the context of changes in vascular permeability leading to a greater capillary leakage of albumin (60), among other factors, such as the underlying nutritional status, nitrogen balance, or renal replacement therapy (61,62), has been associated with poor outcomes in patients COVID-19 (63-65). Data on changes in albumin levels over the course of severe COVID-19 are limited, as discussed below.

Albumin kinetics in patients critically ill with COVID-19 compared with patients critically ill with sepsis or other causes have recently been studied (66). Albumin levels were found to decrease rapidly following admission in patients with COVID-19 regardless of outcome, whereas the recovery in albumin levels predicted clinical improvement in this cohort. Notably, the decline, nadir and recovery of albumin levels in patients with COVID-19 were more pronounced compared with those patients with illnesses of non-COVID-19 etiology (66). In another study (26), in non-survivors, the serum albumin values were below the normal reference range and were significantly lower in non-survivors than in survivors, suggesting that a decline in liver synthesis function may be a key factor for COVID-19-associated mortality. However, elsewhere (20), albumin levels were found to decrease over time in all patients over the first 7 days.

## 4. Comments

The present narrative review provided a summary of the currently available data regarding the temporal evolution of the distinctive characteristic hematological and biochemical parameters, routinely measured in patients admitted to the ICU due to COVID-19. A total of 25 studies were identified, providing trajectories of laboratory parameters during the stay in the ICU, for a follow-up period up to 21 days after ICU admission and/or at ICU discharge.

The following two main observations can be elicited: First, almost all laboratory tests, routinely used on ICU admission and thereafter, were reported to be persistently abnormal over the follow-up period, although they progressively tended to normalize in patients with less severe disease. Second, in the majority of the studies identified, the temporal evolution of the majority of the laboratory parameters was distinct between survivors and non-survivors at certain time points, indicating its clinical importance in prognosis and clinical outcomes.

Certain issues that emerged from the studies included in the present narrative review should be commented on: First, it is worth noting, that in almost all the studies identified, patients in the ICU who were not ill with COVID-19 were not included as a control group. As a result, comparisons of the laboratory trends are mainly focused between survivors and non-survivors, whereas data reporting comparisons between laboratory trends in COVID-19 and infection of other etiology are lacking. In only two studies (49,66), for the examination of the evolution of coagulation, fibrinogen and endothelial function biomarkers (49), and the kinetics of serum albumin in patients critically ill with COVID-19(66), control groups were included.

In the same context, although lymphopenia has been described as a hallmark of severe COVID-19, severe and persistent lymphopenia, is regularly described in patients with sepsis admitted to the ICU (67,68). Of note, a recently reported screening tool for sepsis was based on lymphocyte count, international normalized ratio (INR) and the procalcitonin level (69). Accordingly, COVID-19, as a cause of viral sepsis (70), can induce marked and persistent alterations in lymphocyte counts. Therefore, to date, differences in the dynamic profile of laboratory parameters over time between COVID-19 and other viral infections causing critical illness remain inconclusive.

Second, although a low incidence of co-infections has been reported in patients with COVID-19 at the time of hospital admission (71), multiple studies have confirmed a high incidence of ICU-acquired infections, both bacterial (71,72) and fungal (73), which complicate the clinical course and contribute to high morbidity and mortality rates. Therefore, the laboratory trends provided by the aforementioned studies, which have been included in the present review, need to be interpreted with caution, since their results may have been influenced by the development of secondary infections and not by the SARS-CoV-2 infection per se.

Third, over the course of the pandemic, the clinical practice after the first wave, has evolved with the use corticosteroids (57), tocilizumab (58) or other medications (74). Such treatment strategies may have affected the biomarkers trajectories in patients with COVID-19 throughout hospitalization. Indeed, a large multicenter study (56) demonstrated that the characteristics and disease course of patients critically ill with COVID-19 continuously altered throughout the pandemic, with distinctly different clinical and laboratory parameters than those at the onset of the pandemic. Indicatively, a more pronounced decrease in CRP levels over the duration of stay in the ICU and a concomitant increase in leukocyte numbers and, specifically, neutrophil counts at the later stages of the pandemic have been shown, possibly reflecting the systematic initiation of corticosteroids and/or tocilizumab (56).

Certain limitations of the present narrative review should be mentioned. First, a full coagulation profile, including prothrombin time, INR or other endothelial-associated markers of sepsis-associated coagulopathy (75), were not discussed since the majority of the included studies, as shown in Table I, mostly used serial measurements of PLT counts and D-dimer levels.

Second, temporal trends of other laboratory markers of interest, such as ferritin, interleukin-6 and other cytokines, also characteristic of COVID-19, were not included as the selection of the laboratory parameters was based on those measured routinely as part of the daily clinical practice, across different geographic areas, and not for research purposes.

Third, information related to patients' comorbidities, vaccination status, type of virus and the evolution of illness severity along with the laboratory changes was included in the present review, as the majority of the included articles focused exclusively on the temporal trends of laboratory parameters. However, owing to comparisons between survivors and not survivors, the evolution of the changes in laboratory parameters reflects the different disease severity over time and the association of these parameters with clinical outcomes.

## 5. Conclusion

In conclusion, the temporal evolution of abnormalities of most characteristic routine laboratory parameters in patients critically ill with COVID-19, following admission to the ICU, highlights the essential contribution of laboratory medicine to the pandemic. Persistently abnormal values in laboratory parameters over the course of stay in the ICU in both survivors and non-survivors have been demonstrated. Moreover, in the majority of studies included, the dynamic changes in blood cell counts and biochemical parameters presented significant differences over time and/or between survivors and non-survivors in certain time points, over the course of the disease. Therefore, evaluating the patterns of the temporal changes of certain laboratory parameters may prove to be useful in estimating the severity of the disease and predicting the outcomes of patients with COVID-19 in the ICU. However, further research is required to compare the trends in laboratory parameters in patients with COVID-19 with those of patients with infections of other etiologies.

## Figures and Tables

**Table I:** Clinical studies including an analysis of the temporal evolution of routine laboratory parameters in critically ill patients admitted to the ICU due to COVID-19^a^.

Authors (year of publication)	Setting	No. of patients	Laboratory indices	Follow-up period	Main findings/comments	(Refs.)
Banno *et al* (2021)	Japan	24	WBC, CRP, NLR, D-dimer, LDH	All days of illness	WBC, NLR and CRP followed a similar trend and peaked on day 10 of illness; LDH levels were elevated in the initial phase of illness and subsequently decreased; D-dimer levels were more likely to increase after day 20 of illness/	(15)
Aladağ *et al* (2021)	Turkey	50	WBC, CRP, fibrinogen, D-dimer, lymphocyte and PLT count	Days 0 and 7	Both admission and 7th day lymphocyte count was lower in non-survivors compared to survivors; CRP levels declined in survivors.	(16)
Zheng *et al* (2020)	China	34	WBC, neutrophil, lymphocyte and PLT counts, D-dimer, CRP, LDH	Days 0,1,3,5,7 and on day of discharge	WBC and neutrophils were elevated during the whole period; they normalized only on discharge day in patients without MV; lymphocytes were persistently less than the normal range, especially in patients under MV; D-dimer levels were persistently high especially in patients under MV; CRP levels were persistently higher than normal except on the day of discharge for patients without MV.	(17)
Wang *et al* (2020)	China	33	WBC, neutrophil and lymphocyte counts, D-dimer	Day 1 to day 19 after the onset of the disease	Marked lymphopenia and higher WBC and neutrophil counts, as well as D-dimer levels over time in non-survivors.	(18)
Chen *et al* (2020)	China	548	Hematologic and immunologic biomarkers	Admission, mid- and end of hospitalization	Lymphocytes and PLTs exhibited an increasing trend in survivors, and lower levels or decreasing trend in non-survivors; neutrophils, and D-dimer and CRP levels were higher and exhibited an upward trend in non-survivors, whereas they were stable or exhibited a downward trend in survivors.	(19)
Wendel-Garcia *et al* (2020)	Europe	639	WBC count, NLR, PLT count, d-dimer, CRP, LDH, albumin	First 7 days	Persistently high WBC, NLR, D-dimer and LDH levels; rising CRP dynamics in non-survivors; PLTs increased in all patients, with ICU survivors presenting consistently higher counts; albumin. levels decreased over time in all patients	(20)
Zanella *et al* (2021)	Italy	1260	Neutrophils, lymphocytes and PLT count, NLR, D-dimer, CRP	Daily during the ICU stay	Both daily values and trends of CRP, lymphocytes, NLR and PLTs were associated with survival; the trend in CRP levels exhibited a higher association with survival compared to the daily values; D-dimer levels decreased over time in both groups, but with no association with survival.	(21)
Chen *et al* (2021)	USA	251 (out of 1,252) ICU patients	WBC and lymphocyte count, D-dimer, CRP, LDH	Daily, until the day of death or discharge	A downward trend in CRP to normal values was observed in survivors; its dynamic trend over time, rather than single values was predictive of outcome; temporal fluctuations of most variables were markedly higher in non-survivors compared to survivors.	(22)
Xie *et al* (2020)	China	733	Lymphocyte count, CRP, D-dimer, LDH	Days 1, 3, 7 and 14	CRP and LDH levels significantly decreased in survivors, but remained higher in non-survivors; D-dimer levels were relatively stable, although significantly higher in non-survivors; dynamic changes in CRP, LDH and D-dimer levels, but not in the lymphocyte count, over time, differed significantly different between survivors and non-survivors.	(23)
Montrucchio *et al* (2021)	Italy	57	CRP, LDH, lymphocytes, D-dimer	Days 2,3,7 and 14	Time-dependent analysis of CRP and LDH levels revealed significant differences between survivors and non-survivors, and over time; lymphocytes differed only between survivors and non-survivors; D-dimer levels did not exhibit any difference between the groups and over time.	(24)
Zhou *et al* (2020)	China	195	WBC, neutrophil, lymphocyte and PLT counts	Days 1,3,7,14, and 21	Over the course of the disease, lymphopenia and thrombocytopenia were higher in non-survivors	(25)
Ouyang *et al* (2020)	China	107	WBC, neutrophil, lymphocyte, PLTs, D-dimer, fibrinogen, LDH, albumin	Daily	Increasing trends in LDH, WBC and neutrophil count; decreasing trends in lymphocyte count and albumin levels in non-survivors.	(26) .
Bolondi *et al* (2020)	Italy	31	Lymphocyte count, D-dimer	14 days	Lymphopenia was severe and constant, with a nadir on day 2 of ICU stay; D-dimer levels exhibited a non-significant tendency to increase after ICU admission.	(27)
Ye *et al* (2020)	China	349 (44 with respiratory failure)	NLR, D-dimer	All days of hospitalization	Higher values of D-dimer and NLR in deceased patients than in survivors.	(36)
Barret *et al* (2021)	USA	3,915 (1,415 with critical illness)	PLT count	28 days	PLT count increased during the course of the disease and peaked at approximately 8 days of hospitalization; it was persistently lower in critical illness and in non-survivors.	(39)
Liao *et al* (2020)	China	380 (86 with critical illness)	PLT count, fibrinogen, D-dimer	25 days	PLT count decreased in non-survivors compared with survivors throughout the clinical course; D-dimer levels increased for non-survivors compared with survivors towards the end of the study period; fibrinogen levels decreased over time in both survivors and non-survivors.	(41)
Corrêa *et al* (2020)	Brazil	30	PLT count, fibrinogen, D-dimer	Days 0,1, 3, 7 and 14	PLT count increased from day 0 to day 14; lower counts were observed over time in patients with a SOFA score >10 compared to those with a SOFA score ≤10; fibrinogen levels increased in both groups; highest values observed on day 1 in patients with a SOFA score ≤10 and on day 3 in those with a SOFA score >10; D-dimer levels over time were higher than normal range in both groups.	(42)
Spadaro *et al* (2021)	Italy	31	D-dimer	Days 1, 7 and 14 after ICU admission	D-dimer levels did not differ at ICU admission between survivors and non-survivors, but there was an increase over time in non-survivors	(44)
Pavoni *et al* (2020)	Italy	40	PLT count, fibrinogen, D-dimer	Days 0, 5 and 10	Fibrinogen values significantly decreased from day 0 to day 10; no other significant change.	(45)
Dujardin *et al* (2020)	The Netherlands	127	Platelet count, CRP, D-dimer, fibrinogen	Daily for the first 20 days or until discharge	Patients were stratified on the base of VTE development. D-dimer levels significantly increased on days 4 and 8 in the VTE group compared to the non-VTE group; CRP levels were significantly higher in the VTE group up to day 16; fibrinogen levels and the PLT count persistently increased in all patients.	(47)
Juneja *et al* (2021)	Canada	14	D-dimer, fibrinogen	10 consecutive days	D dimer and fibrinogen trajectories were not associated with outcomes.	(49)
Oskarsdottir *et al* (2022)	Iceland	59 (out of 571) admitted to the ICU or did not survive	WBC, neutrophil and lymphocyte count, CRP	For 22 days since the onset of symptoms	Patients who were either admitted to the ICU or did not survive had early and persistent separation of lymphocyte count and CRP levels, as well as higher WBC and neutrophil counts.	(54)
van Oers *et al* (2021)	The Netherlands	105	CRP	Daily for 7 days or until discharge or death	Time-dependent analysis of CRP levels; no differences found between survivors and non-survivors, but significance over time	(55)
Zacharias *et al* (2022)	UK	214	CRP	Daily for the first 12 days	A reduction in CRP levels was found in the first 3 days of dexamethasone treatment, which subsequently increased in non-survivors; by contrast, CRP levels decreased and remained low in survivors and non-survivors who received tocilizumab.	(59)
Su *et al* (2021)	USA	308	Albumin	Daily, up to 30 days	Rapid albumin loss followed by albumin stabilization or improvement; albumin recovery predicted clinical improvement.	(66)

^a^In some of the included studies, more laboratory parameters were examined; only those of interest (i.e., those included in the present narrative review, on the basis of their routine use) are presented in the table. WBC, white blood cell; PLT, platelet; CRP, C-reactive protein; LDH, lactate dehydrogenase; NLR, neutrophil-to-lymphocyte ratio; ICU, intensive care unit; MV, mechanical ventilation; VTE, venous thromboembolism; SOFA, sequential organ failure assessment.

## Data Availability

Not applicable.
